# Identification and Venom Characterization of Two Scorpions from the State of Chihuahua Mexico: *Chihuahuanus coahuliae* and *Chihuahuanus crassimannus*

**DOI:** 10.3390/toxins15070416

**Published:** 2023-06-27

**Authors:** Carolina Alvarado-Gonzalez, Herlinda Clement, Lourdes Ballinas-Casarrubias, Angelica Escarcega-Avila, Ivan Arenas-Sosa, Karla Sofia Lopez-Contreras, Fernando Zamudio, Gerardo Corzo, Gerardo Pavel Espino-Solis

**Affiliations:** 1 Traslational Research Laboratory, Facultad de Medicina y Ciencias Biomédicas, Autonomous University of Chihuahua, Circuito Universitario s/n, Campus II, Chihuahua 31125, Mexico; p274265@uach.mx (C.A.-G.); a342103@uach.mx (K.S.L.-C.); 2 Facultad de Ciencias Quimicas, Autonomous University of Chihuahua, Circuito Universitario s/n, Campus II, Chihuahua 31125, Mexico; mballinas@uach.mx; 3 Instituto de Biotecnología—UNAM, Universidad Nacional Autónoma de México, Av. Universidad 2001, Col. Chamilpa, Cuernavaca 62210, Mexico; herlindaclement@gmail.com (H.C.); ivan@ibt.unam.mx (I.A.-S.); zam@ibt.unam.mx (F.Z.); gerardo.corzo@ibt.unam.mx (G.C.); 4 Veterinary Sciences Department, Autonomous University of Ciudad Juarez, Ciudad Juarez 32310, Mexico; maria.escarcega@uacj.mx; 5 Laboratorio Nacional de Citometría de Flujo, Facultad de Medicina y Ciencias Biomédicas, Autonomous University of Chihuahua, Circuito Universitario s/n, Campus II, Chihuahua 31125, Mexico

**Keywords:** Chihuahua, *C. crassimanus*, *C. coahuliae*, scorpion venom, insecticidal, antimicrobial

## Abstract

Chihuahua is the largest state in Mexico. The ecosystem of this region is composed of large area of bushes, forests, and grasslands, which allows for a specific diversity of fauna; among them are interesting species of non-lethal scorpions. Most of the Chihuahuan scorpions have been previously morphologically and molecularly described; however, this manuscript could be the first to describe the composition of those venoms. This work aimed at the collection of two scorpion species from the region of Jiménez (Southwest of the State of Chihuahua), which belong to the species *Chihuahuanus cohauilae* and *Chihuahuanus crassimanus;* the two species were taxonomically and molecularly identified using a 16S DNA marker. Reverse-phase high-performance liquid chromatography (RP-HPLC) of *C. coahuilae* and *C. crassimanus* venoms allowed the identification of three fractions lethal to mice. Additionally, three fractions of each scorpion displayed an effect on house crickets. In the end, three new fractions from the venom of *C. coahuilae* were positive for antimicrobial activity, although none from *C. crassimanus* venom displayed growth inhibition. Despite being a preliminary study, the venom biochemical analysis of these two uncharacterized scorpion species opens the opportunity to find new molecules with potential applications in the biomedical and biotechnological fields.

## 1. Introduction

Scorpions represent a species with more than 400 million years of evolution [1,2]. They can be found almost anywhere in the world, except in the North and South Poles [3,4]. In addition, they can be divided according to their geographic location into Old and New World scorpions and by their medical importance into scorpions that are potentially dangerous to humans and those that are not [5]. Nevertheless, of the 2231 species that have been identified only 30 of them are considered dangerous to humans [6,7]. Scorpion venoms contains mainly a bulk of ion channel toxins (Na^+^, K^+^, Cl^−^, and Ca^2+^), mucopolysaccharides, and enzymes [8,9,10,11]. This massive source of compounds is commonly used to disrupt biochemical and physiological processes in target organisms [12].

The scorpion ion channel toxins are interesting small molecules reticulated by disulfide bridges that fold in different types of structures such as the cysteine-stabilized-helix and -sheet (CSab), the disulfide-directed beta-hairpin (DDH), and the inhibitor cystine knot (ICK) [11]. These compact structures have a diverse potential of pharmacological applications with biomedical interest and biotechnological applications, such as pain inhibitors, antimicrobial peptides, immunosuppressants, anticancer, insecticidal toxins, etc. [13,14,15,16].

Today, current health scenario concerning the challenges of antibiotic resistance in bacteria and the lack of adequate and little or no harmful side effects treatments against degenerative diseases and cancer, could be a skillful opportunity for scorpion toxin prospectors to mine newly identified species of scorpion venoms to find the appropriate molecules for selected targets.

In this work, we present a partial picture of the characterization of the venom of two scorpion species found in the State of Chihuahua. Peptide fractions which are lethal to mammals, insecticidal, and antimicrobial were identified. However, further experiments must be conducted to discern the primary structure of such peptides.

## 2. Results

### 2.1. Scorpion Collection Site

**Figure 1:**
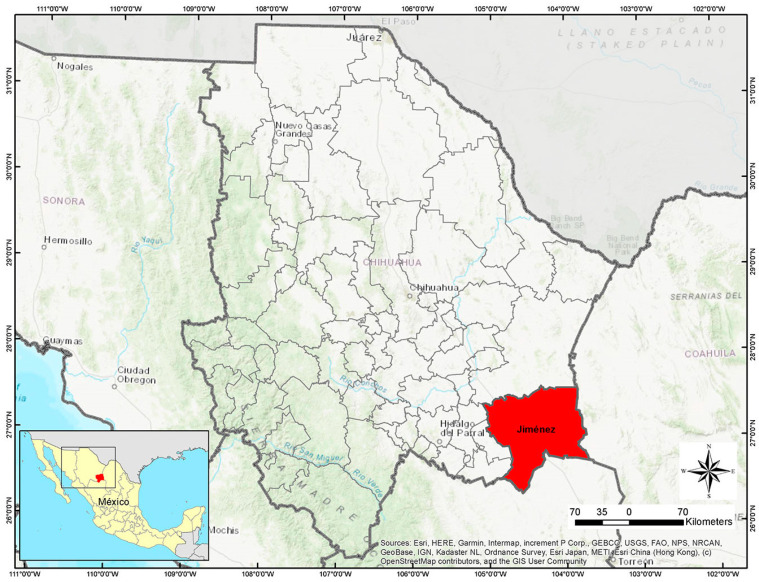
Scorpion collection. Specimens were collected around Jiménez in the south of the State of Chihuahua, Mexico (27.114384, −104.879348), map generated by authors using QGIS Software v 2.18.12.

The State of Chihuahua is situated in the Northwestern region of Mexico, bordering New Mexico and Texas in the north. The Mexican States of Coahuila in the east, Durango in the south, Sinaloa in the southwest, and Sonora in the west (Figure 1). The scorpion collection was carried out around the City of Jiménez, situated in the southeast of the State of Chihuahua (Figure 1). From June to September 2022, specimens were collected at night using ultraviolet light lamps. Scorpions were found mainly under stones, wood, and near bushes. Individual containers for scorpions with water and food were available.

### 2.2. Classification Molecular and Taxonomic

**Figure 2:**
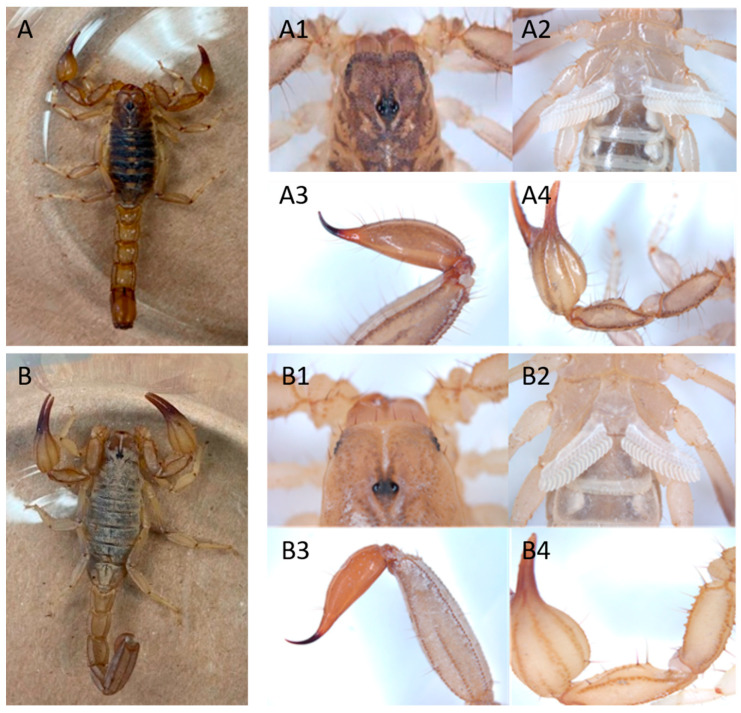
Taxonomic classification. (**A**). *Chihuahuanus coahuilae*. (**A1**) Carapace, dorsal aspect, showing one pair of central eyes and two pairs of lateral eyes. (**A2**) Ventral photo of a scorpion showing its pectins. (**A3**) Venom gland (telson, lateral aspect). (**A4**) Pedipalp of a scorpion covered with sensory hairs. (**B**) *Chihuahuanus crassimanus*. (**B1**) Carapace, dorsal aspect, showing one pair of central eyes and two pairs of lateral eyes. (**B2**) Ventral photo of a scorpion showing its pectins. (**B3**) Venom gland (telson, lateral aspect). (**B4**) Pedipalp of a scorpion covered with sensory hairs.

**Figure 3:**
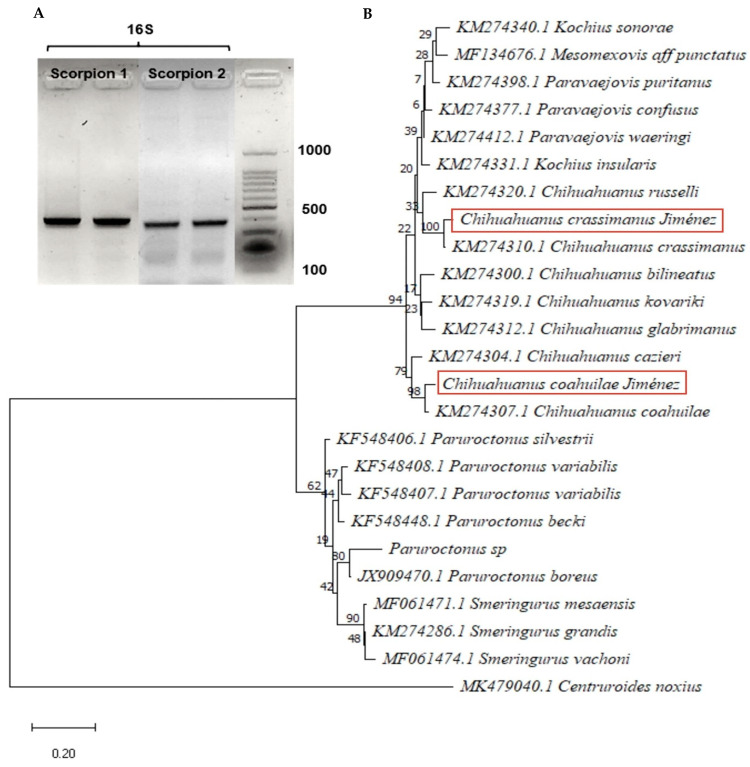
Molecular identification and phylogenetic analysis of the 16S gene. (**A**) PCR amplification products; 12S rRNA gene (450 bp), 16S rRNA gene (400 bp), COI gene (540 bp). The primers were reported by Borges et al. (2010) [17]. (**B**) Molecular phylogenetic analysis of the 16S rDNA mitochondrial gene using the maximum likelihood (ML) method generated with the Tamura 3-parameter Model with Gamma distribution (T92+G) with 100 bootstrap replicates. The tree with the highest logarithmic probability is displayed. The percentage of trees in which the associated taxa clustered is shown next to the branches. Each sequence is indicated by its GenBank accession number. The boxes indicate the sequences obtained in this project.

The morphology and DNA extraction were the initial basis for the molecular identification of the two scorpions. The morphological analysis consisted of observing different taxonomic parts of the specimens, referring to the characteristics reported for the species belonging to the *Vaejovidae* family; the morphology observed in this species matches data previously reported [3] (Figure 2). The 16S rDNA marker was used for scorpion identification (Figure 2), which allowed the obtention of the nucleotide sequence. Further search in the NCBI database (Basic Local Alignment Search Tool https://blast.ncbi.nlm.nih.gov/ accessed on 12 April 2023), allows the selection of DNA sequences to perform a phylogenetic analysis. This was performed using the MEGA11 software, according to the Bayesian Information Criterion (BIC) score. The phylogenetic tree was built with the Tamura 3-parameter nucleotide substitution model with Gamma distribution (T92+G) and 100 bootstrap replicates. The red box shows the species of interest in this study, among 98 matches with the species reported in the database as *Chihuahuanus coahuilae* with the identifier KM274307.1 (Figure 3). On the other hand, *Chihuahuanus crassimanus* presents an exact match with the sequence reported in the database with the identifier KM274310.1 (Figure 3).

### 2.3. Mice and Cricket Biological Activity

**Figure 4:**
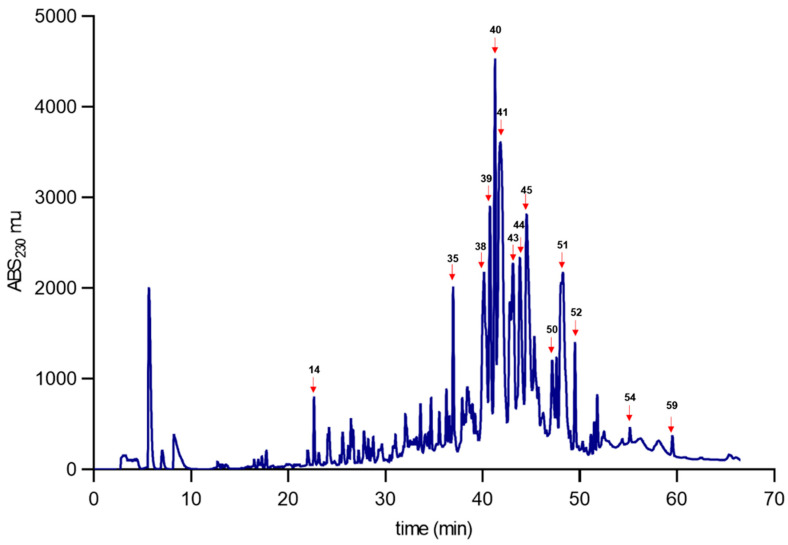
Venom separation analysis of *Chihuahuanus coahuilae*. The venom obtained from scorpions was separated by RP-HPLC, using a C18 column in a 0 to 60% acetonitrile linear gradient, in 0.1% TFA. Selected fractions marked with numbers and red arrows were further analyzed by mass spectrometry.

**Figure 5:**
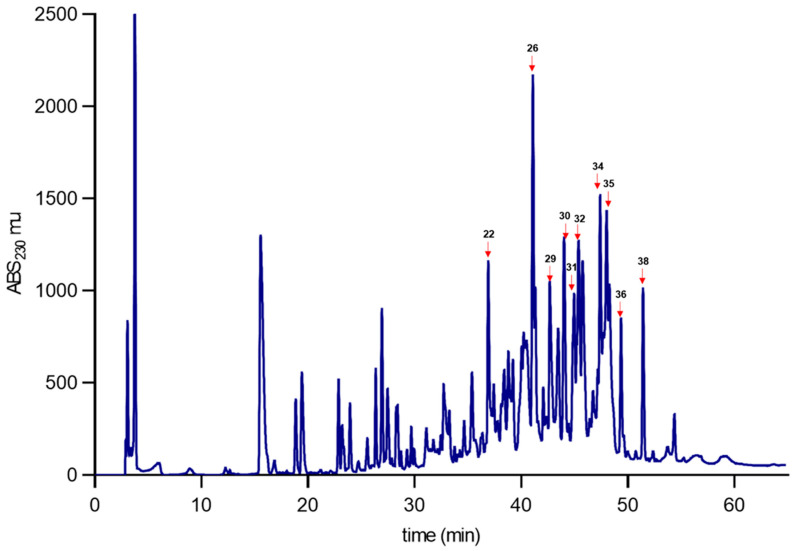
Venom separation analysis of *Chihuahuanus crassimanus*. The venom obtained from scorpions was separated by RP-HPLC, using a C18 column in a 0 to 60% acetonitrile linear gradient, in 0.1% TFA. Selected fractions marked with numbers and red arrows were further analyzed by mass spectrometry.

The venoms of *C. coahuilae* and *C. crassimanus* were separated using reversed-phase (RP) chromatography. Sixty-two and forty-seven fractions from *C. coahuilae* and *C. crassimanus* were collected, respectively (Figure 4 and Figure 5). The molecular masses of the selected fractions were evaluated by electrospray ionization mass spectroscopy (ESI-MS) resolving a partial fingerprint of the main components (Table 1 and Table 2). The most abundant fractions (labelled with numbers) were tested for toxicity on CD-1 mice and insects to evaluate (Table 1 and Table 2). Fractions 14, 39, and 43 (*C. coahuilae*) and fraction 29 (*C. crassimanus*) were lethal at a concentration of 2 µg/20 g when injected intracranially into mice. Furthermore, fractions 43, 44, 45, and 51 (*C. coahuilae*) and fractions 29, 30, and 36 (*C. crassimanus*) were evaluated for insecticidal activity using house crickets as an insect model. In this assay, fractions 43, 45, and 51 from *C. coahuilae* had a lethal effect at a dose of 10 µg per cricket, and fraction 44 resulted in paralysis of the insect, but it was not lethal. On the other hand, fractions 29, 30, and 36 from *C. crassimanus* were paralytic at 10 µg per cricket (Table 1 and Table 2).

### 2.4. Antimicrobial Activity

**Figure 6:**
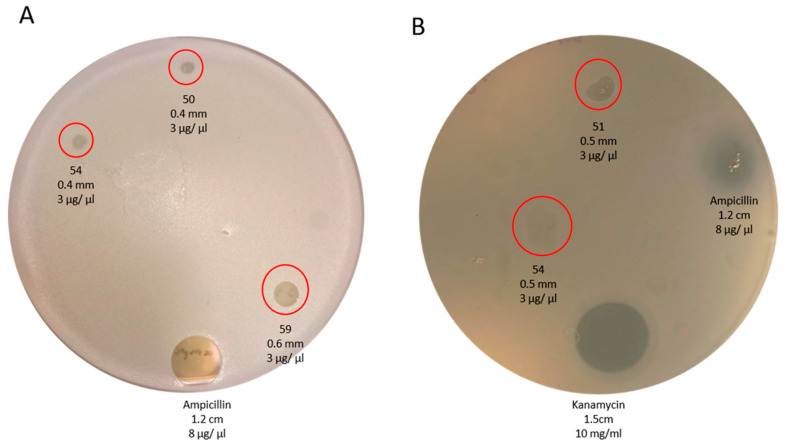
Surveillance of antimicrobial activity in venom fractions obtained from *Chihuahuanus coahuilae.* Selected HPLC fractions were assayed at 3 µg/µL in (**A**) Gram-positive *Staphylococcus aureus* ATCC 29213 and (**B**) Gram-negative *E. coli* ATCC 25922. Red circles display the inhibition halo of the selected fractions. Ampicillin and Kanamycin as a positive control, and H_2_O negative control.

Concerning the antimicrobial activity, fractions 50, 51, 54, and 59 from the venom of *C. coahuilae* showed inhibition of the bacteria growth on LB agar plates; however, none of the fractions from the venom of *C. crassimanus* was positive for antimicrobial activity. Fraction 51 inhibited just *E. coli*. Fraction 54 inhibited both *E. coli* and *S. aureus*, and fractions 50 and 59 were active only against *S. aureus*. Each fraction from the venom of *C. coahuilae* were assayed at a fixed concentration of 3 µg/mL (Table 3). Ampicillin and Kanamycin were used as a positive control at a concentration of 8 and 10 µg/µL, respectively, and dH_2_O was used as a negative control. Bacteria were incubated with venom fractions for 18 h at 37 °C and inhibition zones were observed (Figure 6).

## 3. Discussion

In recent years, progress has occurred in the study of the diversity of Mexican scorpion species. The diversity of the State of Chihuahua has provided conditions for the *Vejovidae* scorpions. Even though the sting of scorpion species found in Chihuahua does not represent a medical problem, the characterization of Chihuahuan scorpion venoms can provide access to a novel repertoire of molecules with potential application in biomedicine [15,17]. The morphological characteristics of the species *Chihuahuanus coahuilae,* found in the southeast of the State of Chihuahua, correspond to the description made by Williams in 1968, where he described *Vaejovis* species linked to the ones found in the State of Coahuila. The general classification of the specimens found were medium-sized species with mature females attaining larger body sizes than males. Body base color has been reported as dirty yellow; one pair of dark stripes on the dorsum of mesosoma; carapace with irregular dusky markings; two pairs of dark stripes underlying inferior keels (darkened or granular raised linear ridges) of metasoma. Pedipalp hands lacking granulation, keels, and pectinal teeth variating from 13 to 16 in females and 17 to 21 in males. Later this species would be redefined by González-Santillán, classified as *Chihuahuanus* genus.

The morphological data concerning *Chihuahuanus crassimanus* is found in this manuscript. The difficulties to identify species based on morphological techniques, as well as the small number of studies, are some of the reasons why the use of molecular markers can provide a tool for precise assessment of the diversity of species found in the State of Chihuahua [2,18]. So, in this work, the 16S rDNA was successfully differentiated between genera, and the nucleotide sequences were well clustered on the species level (Figure 3). González-Santillán and Prendini classified various species, including the species found in the southeast of the State of Chihuahua, into the *Syntropinae* family. The species found in Chihuahua belongs to the *Vaejovidae* family, which is one of the families with the larger number of species in Mexico [19,20], the scorpions belonging to this family are not considered of medical importance. However, the diversity of components in them has been proven to be a natural source of compounds with possible therapeutic applications [7].

The phylogenetic tree shows that the genetic marker can differentiate the species with reliability. Based on the report of González-Santillán, which describes six new genera of the species of the *Syntropinae Kraepelin* subfamily, the species *Chihuahuanus coahuilae* and *Chihuahuanus crassimanus* shows nearly the same phylogenetic results.

The severity of scorpion envenomation is related to the existence of neurotoxins in the venom [21]. They can block or modify the functioning of their target ion channels in excitable cells, resulting in autonomous excitation. Toxins that affect sodium channels have 60 to 70 amino acids and four disulfide bridges [22], while toxins that affect potassium channels have 30 to 40 amino acids and 3 or 4 disulfide bridges [23]. In addition to these two main groups, other toxins affect calcium and chloride channels [19,24]. Some toxins are selective for insects, and others have antibacterial activity, which gives them a potential use in the development of bioinsecticides and in the therapeutic control of pathogenic bacteria [25].

A preliminary characterization of the biochemical composition of the venom from *C. coahuilae* and *C. crassimanus* was performed. Some fractions were lethal to mammals, and others lethal and toxic to insects. Other fractions had antimicrobial activities.

Hadrurin was the first antimicrobial peptide isolated from the Mexican scorpion *Hadrurus aztecus*, a 41-amino acid peptide that could inhibit the growth of the bacteria *Salmonella thyphi*, *Klebsiella pneumoniae*, *Enterococcus cloacae*, *Pseudomonas aeruginosa*, *Escherichia coli,* and *Serratia marscences* [26,27]. Other antimicrobial peptides isolated from the Mexican scorpion *Vaejovis mexicanus* are VmCT1 and VmCT2 [28,29]. These peptides are 13 amino acids long and were synthesized chemically including the amidation at their N-terminal region.

On the other hand, the synergistic effect of commercial antibiotics with the peptides La47 and Css54, isolated from the venom of the spider *Lachesana* sp., and the scorpion *Centruroides sufussus*, respectively, were evaluated. When contrasting its biological activity with the Pin2 peptide isolated from the venom of the *Pandinus imperator* scorpion, uncovering that La47 has a lower hemolytic and antimicrobial activity in contrast to Css54 and Pin2. When La47 and Pin2 were assayed in the presence of commercial antibiotics, the best combination was obtained by mixing La47 and Pin2 with the antibiotic chloramphenicol, streptomycin, and kanamycin [30]. The presence of molecules with antimicrobial activity was detected in the *Chihuahuanus coahuilae* venom when it was assayed on Gram-positive and Gram-negative strains (Figure 4), further data also is summarized at supplementary material (Figure S14).

The presence of molecules with toxicity effects in mammals and insects may suggest the possibility that the venom of these two species could contain toxins that target ion channels. Based on their biological effects and a comparison of their molecular weight, it could be theorized that these compounds may be toxins acting on voltage-gated sodium (Nav) channels and voltage-gated potassium ion channels (Kv). There is information that the toxins affecting Nav have a molecular weight ranging from 6500 to 7800 Da and could be toxic to mammals and insects [8,16]. Based on this data, it could be assumed that fraction 43 (RT 43.1) of *C. coahuilae* (6559.1–6736.1 Da) and fraction 29 (RT 43.7) of *C. crassimanus* (6709.4–6747.2 Da), which showed toxic effects in both mammals and insects (Supplementary Tables S1–S3), may belong to this group of toxins; however, this factions have to be repurified and assess for toxicity since two molecular weights appear in each peak, which may suggest the presence of two different peptides. It is valuable to highlight fractions 44 (RT 43.6) of *C. coahuilae* (6643.7–6616.4 Da) and 30 (RT 44.3) of *C. crassimanus* (6643.7 Da) since both fractions were toxic to crickets and such fractions share peptides with similar molecular weights, they may have similar primary structure (Supplementary Tables S1–S3). However, fraction 44 must be purified, this data opens the question about the importance of this putative insect-toxin in different scorpions, and further experiments must be performed to uncover this biological coincidence.

On the other hand, voltage-gated potassium ion channels (Kv) toxins that have a molecular weight average ranging from 3000 to 4500 Da. are organized into four different families: *α*-, *β*-, *γ*-, and *κ*-KTx, which can act in distinct ways such as blocking, modulating activity, or interfering with the regulation of potassium flux [31]. Therefore, the interaction of scorpion venom toxins with Kv can have detrimental effects on different systems in the body. For example, it can affect the function of the nervous system, causing symptoms such as pain, muscle spasms, seizures, and alterations in signal transmission between nerve cells [32]. An example of such toxins derived from the venom of the scorpion *Vaejovis mexicanus smithi* is the toxin Vm24 (3864 Da.), and it can be presumptive that fraction 14 (3540.8 Da) of *C. coahuilae* could be a KTx due to its action in mammals [33]. Additional data are also in supplementary materials (Figure S15).

Figure S1 (supplementary material) shows the Reverse-phase high-performance liquid chromatography (RP-HPLC) of *C. coahuilae* and *C. crassimanus* venoms in an overlapped format is able to identify some shared peaks among the species (Supplementary Tables S1–S3) summarizes retention times and molecular weight.

## 4. Conclusions

The venoms of *C. coahuilae* and *C. crassimanus* were separated using reversed-phase (RP) chromatography. Sixty-two and forty-seven fractions from *C. coahuilae* and *C. crassimanus* were collected, respectively. Four fractions: 14, 29, and 43 (*C. coahuilae*) and one fraction 29 (*C. crassimanus*), displayed lethal outcomes when injected intracranially into mice. In addition, venoms were evaluated for insecticidal activity using house crickets as insect model fractions: 43, 45, and 51 from *C. coahuilae* had a lethal effect and fraction 44 resulted in insect paralysis. On the other hand, fractions 29, 30, and 36 from *C. crassimanus* were paralytic effects. Regarding the antimicrobial activity, fractions 50, 51, 54, and 59 from the venom of *C. coahuilae* showed inhibition of the bacteria growth. No antimicrobial activity was detected in the venom of *C. crassimanus.*

To wrap up, *C. coahuilae* and *C. crassimanus* venoms have revealed new information about their composition and potential biotechnological applications. Moreover, studies have shown molecules isolated from scorpion venom can be used to develop new drugs for autoimmune diseases and neurological disorders. However, more research is needed to fully understand the complex mechanisms of scorpion venom from the state of Chihuahua. Venom purification and fraction surveillance have shown that biological activity work is in progress to inspect these novel compounds that may have potential uses in biomedicine. Nonetheless, the study of scorpion venom represents an exciting and promising avenue for drug development and has the potential to make a significant impact on human health.

## 5. Material and Methods

### 5.1. Scorpion’s Collection and Maintenance

Specimens were collected from the southeast region of the State of Chihuahua (27.114384, −104.879348) during the months of June-September of the year 2022. Scorpion field collection was granted by SEMARNAT (No. SPARN/DGVS/02544/23). Scorpions were kept in captivity under standard temperature conditions and supplied with water. They were maintained in an individual container until venom extraction protocol. The guidelines set out according to the Official Mexican Standard for the use of laboratory animals (NOM-062-ZOO-1999) were followed. Furthermore, animal handling was approved by the research and ethics committee at the Faculty of Medicine and Biomedical Sciences No. CI-068-19, which allows animal experimentation.

### 5.2. DNA Extraction, Amplification, and Sequencing

Disruption of the cellular structure to create a lysate was carried out by macerating the mesosoma of a scorpion, proteinase K (Thermo Fisher Scientific, Waltham, MA, USA) and phenol β-mercaptoethanol (Sigma-Aldrich, St. Louis, MO, USA) were added and centrifuged to separate the soluble DNA from cell debris and other insoluble materials. A process of adding different compounds such as isoamyl alcohol (Sigma-Aldrich, St. Louis, MO, USA), sodium acetate (Sigma-Aldrich, St. Louis, MO, USA), and ethanol (Sigma-Aldrich, St. Louis, MO, USA), accompanied by incubation times at different temperatures and centrifugation were carried out to bind the DNA of interest to a purification matrix, ethanol (Sigma-Aldrich, St. Louis, MO, USA) is added to washing proteins and other contaminants away from the matrix and is resuspended in 30–50 µL of sterile water for elution of the DNA.

For 16S rDNA (T4 Oligo, Irapuato, Gto, México) amplifications, the forward primer 5′-CGATTTGAACTCAGATCA-3′ and the reverse primer 5′-GTGCAAAGGTAGCATAATCA-3′ (Borges et al., 2010 [3]) [17]. PCR amplification protocol: 94 °C for 5 min; 30 cycles of denaturing at 94 °C for 30 s, annealing at 48 °C for 30 s, and extension at 72 °C for 30 s; and a final extension phase at 72 °C for 7 min (Applied Biosystems, thermal cycler 2720, Waltham, MA, USA). The PCR reaction was verified by agarose electrophoresis (BIO-RAD, Hercules, CA, USA).

For purification, the NucleoSpin Gel and PCR Clean-up DNA purification kit were used (Macherey-Nagel, Düren, Germany No. Cat 740609.50), following the protocol provided by the supplier. The PCR product was sent to the DNA Synthesis and Sequencing Unit (USSDNA) of the Institute of Biotechnology, Cuernavaca, México, UNAM.

The data obtained from the sequencing of the DNA fragments extracted with the mitochondrial marker were analyzed. A protein Blast (National Center for Biotechnology Information (NCBI), Bethesda. MD, USA) https://blast.ncbi.nlm.nih.gov/Blast.cgi, accessed on 13 April 2023 was performed to the sequence and those with the highest identity percentage were chosen to perform a phylogenetic analysis, this was carried out with the MEGA11 software (Molecular Evolutionary Genetics Analysis version 11, Philadelphia, PA, USA 2021) (https://www.megasoftware.net/, accessed on 13 April 2023) starting with a multiple sequence alignment, followed by the construction of the phylogenetic tree.

### 5.3. Morphological Characteristics Analysis

Taxonomic identification was made with the ZEISS stereoscope using the ZEN 3.4 blue edition software (Carl Zeiss Microscopy GmbH, Göttingen, Germany, 2011). Images of different morphological parts of the specimens, such as the carapace, pedipalps, pectinal teeth, and vesicle/telson were taken [19]. González-Santillán and Prendini described the pedipalp chela fixed finger of *Chihuahuanus*, gen. nov., as consistently exhibited five primary subrows of median denticles and five prolateral denticles in the median denticle row, which was corroborated to *Chihuahuanus coahuilae* (Figure 2(A4)). Also, the diagnosis of the carapace from such as shagreened, comprising coarse, rounded, and fine, scattered granules in most species of *Chihuahuanus*, gen. nov. was observed in the two species (Figure 2(A1,B1)).

### 5.4. Venom Extraction

The venom was obtained by the electrical stimulation method as previously described [20]. Scorpions were placed in a metal grid connected to an electrode, then hold from the fifth metasomal segment. Twelve volts were applied in small pulses (no more than three pulses per scorpion) to the base of the telson with an electrode of opposite polarity. The technique for venom extraction was performed in a controlled environment to ensure the safety of both the extractor and the scorpion and maximizing venom recovery. All the venom was collected with a micropipette tip, subsequently transferred to an Eppendorf tube and stored at −80 °C until use. After extraction, the scorpions were kept isolated and with a feeding schedule until the next extraction.

### 5.5. Fractionation of Scorpion Venoms

Crude venom (2 mg), previously lyophilized (LABCONCO, FreeZone 2.5 Kansas City, MO, USA), was treated as follows: it was suspended in 500 μL of a 0.1% trifluoroacetic solution (Sigma-Aldrich, TFA, St. Louis, MO, USA), then the insoluble material precipitated by centrifugation at 14,000× *g* during 10 min (Eppendorf, centrifuge 5430 R, Hamburg, Germany). The remaining liquid was further fractionated by reverse phase-HPLC (Agilent 1260 Infinity II, Santa Clara, CA, USA) using an analytical C18 column (Supelco Inc. Sigma-Aldrich, Discovery, 25 cm length and 4.6 mm diameter, 5 µm particle size, St. Louis, MO, USA). The column was stored in 0.1% trifluoroacetic acid (TFA). For elution, a gradient was used of acetonitrile (0 to 60%) used as the second solvent 0.1% TFA (Sigma-Aldrich St. Louis, MO, USA). HPLC analysis was for 60 min, mobile phase flow rate 1 mL/min. UV-Vis detector adjusted at 230 nm. The different fractions were collected properly and dried further at high vacuum. The fractions were used for the following toxicological evaluations. Protein concentration was calculated by resuspending for dH_2_O (Thermo Scientific, Waltham, MA, USA) via UV-Vis spectrometry at 280 nm (Thermo Scientific, NanoDrop One C, Waltham, MA, USA). One absorbance unit was equivalent to 1 mg/mL of protein [20].

### 5.6. Mass Spectrometry and N-Terminal Sequencing

Mass spectrometry was performed on the protein fractions previously diluted to 500 pmol/5 μL in a 50% acetonitrile—1% acetic acid. The sample was injected with a Surveyor MS syringe pump delivery system to the mass spectrometer (Thermo Scientific LCQ Fleet ion trap mass spectrometer, San Jose, CA, USA) using a Split mode was used for the eluate at 10 μL/min (5% of the, i.e., 0.5 μL/min). The following parameters were used: 1.5 kV, capillar temperature 150 °C, fragmentation source at 25–35 V, 35–45% normalized collision energy, positive ion-mode, and wide band scanning. Acquisition was attained, average molecular masses ±1 Da, on the Xcalibur Windows NT PC data system (Thermo Scientific, San Jose, CA, USA).

### 5.7. Biological Activities in Mice and Crickets

#### 5.7.1. Mice

The protocol used for assaying the activity of peptides in vivo was followed according to the Institute Committee of Animal Welfare guidelines, keeping the number of animals to a minimum. Male mice (CD-1, 20 g body weight) surveillance by intracranial (ic) injection. Male mice (CD-1, 20 g body weight) were tested by intracranial (ic) injection. Peptide fractions were diluted up to 20–50 μL, at 2 μg/μL concentration, with deionized water (dH_2_O). The injection, with a 10 μL micro-syringe fitted with a glass capillary (Thermo Scientific, Hamilton 700 Microliter Syringes, Rockford, IL, USA), of 1 μL was performed mid-way between the left eye and the left ear (intracranial). Negative controls were carried out with dH_2_O. All assays were performed according to a standard protocol (Organization, W.H., 1981, WHO, Geneva, Switzerland) [34]. Mice were observed for toxicity symptoms up to 24 h.

#### 5.7.2. Insects

House crickets (*Achaeta domesticus*) were injected between the second and third pair of legs, with 5 μL (2 μg/μL concentration) dose of selected fraction dissolved in distillated water. Controls were carried out with dH_2_O, the crickets were observed for up to 120 min. Toxicity evaluations were set as follows: Paralysis was used when the cricket was missing mobility but recovered. Lethal effect was recorded when the crickets died.

### 5.8. Antimicrobial Activity

To test the antimicrobial activity of the venoms and the fractions obtained from scorpion venoms, experiments were performed on the agar plate with the strains *E. coli* ATCC 25922 (Gram-negative) and *S. aureus* ATCC 29213 (Gram-positive). The LB agar medium (Sigma-Aldrich, St. Louis, MO, USA) was heated to liquefaction (25 mL) and kept at 42 °C for 15 min. Then, it was inoculated with 50 µL of the respective bacteria inoculum from a preview overnight culture. The medium with bacteria was placed on a Petri dish (Becton Dickinson, Cuautitlán Izcalli, MX, México) and incubated at room temperature for 15 min. Diluted scorpion fractions were added, and venoms were tested at a concentration of 3 µg/mL at 37 °C overnight; it diffuses into the agar and inhibits the growth of the test microorganism. Diameters of inhibition growth zones were measured. Kanamycin and ampicillin (Sigma-Aldrich St. Louis, MO, USA) were used as a positive control and dH_2_O as a negative control.

## Figures and Tables

**Table 1:** The venom fractions of *Chihuahuanus coahuilae* exhibited distinct experimental molecular masses, as determined through mass spectrometry. These fractions were subsequently evaluated for their biological activity in mice and crickets.

Fraction	Retention Time (min)	Molecular Mass (Da)	Mouse (2 µg)	Cricket (10 µg)
14	22.6	3540.8	Dead	-
35	36	2977.0 3066.1	-	-
38	39.9	7960.3	-	-
39	40.7	8302.3	Dead	-
40	41.2	3302.7	-	-
41	41.6	6559.7	-	-
43	43.1	6559.1 6736.1	Dead	Dead
44	43.6	6643.7 6616.4	-	Paralyzed
45	44.4	2103.9	-	Dead
50	47.7	5212.5 4822.6 4980.7 1888.5	-	-
51	49.1	2157.3 845.1 1377.1	-	Dead
52	49.9	1349	-	-
54	52.2	1364.1	-	-
59	59.5	1536.4	-	-

**Table 2:** The venom fractions of *Chihuahuanus crassimanus* exhibited distinct experimental molecular masses, as determined through mass spectrometry. These fractions were subsequently evaluated for their biological activity in mice and crickets, as well as their antimicrobial activity.

Fraction	Retention Time (min)	Molecular Mass (Da)	Mouse (2 µg)	Cricket (10 µg)
22	37.5	3066.7	-	-
26	41.3	8302.6	-	-
29	43.7	6709.4 6747.2	Dead	Paralyzed
30	44.3	6643	-	Paralyzed
31	45.1	1961.8	-	-
32	45.7	1975.2 1593.7	-	-
34	48.2	2232.6	-	-
35	48.7	9131.2	-	-
36	49.6	2133.3	-	Paralyzed
38	51.6	6176	-	-

**Table 3:** The venom fractions of *Chihuahuanus coahuilae* exhibited different experimental molecular masses, determined by mass spectrometry. These fractions were subsequently assayed for antimicrobial activity (3 µg/µL).

Fraction	Retention Time (min)	Molecular Mass (Da)	Gram Positive	Gram Negative
14	22.6	3540.8	- ^a^	-
35	36	2977.0 3066.1	-	-
38	39.9	7960.3	-	-
39	40.7	8302.3	-	-
40	41.2	3302.7	-	-
41	41.6	6559.7		-
43	43.1	6559.1 6736.1	-	-
44	43.6	6643.7 6616.4	-	-
45	44.4	2103.9	-	-
50	47.7	5212.5 4822.6 4980.7 1888.5	+ ^b^	-
51	49.1	2157.3 845.1 1377.1	-	+
52	49.9	1349	-	-
54	52.2	1364.1	+	+
59	59.5	1536.4	+	-

^a^ The sign “-” means negative activity. ^b^ The sign “+” means positive activity.

## Data Availability

Further data is available on Supplementary Information section.

## Supplementary Materials

The following supporting information can be downloaded at: https://www.mdpi.com/article/10.3390/toxins15070416/s1, Figure S1. Overlapping HPLC profiles of two scorpion venoms. Figure S2. Mass spectrum fraction 14 *Chihuahuanus coahuilae*. Figure S3. Mass spectrum fraction 39 *Chihuahuanus coahuilae*. Figure S4. Mass spectrum fraction 43 *Chihuahuanus coahuilae*. Figure S5. Mass spectrum fraction 44 *Chihuahuanus coahuilae*. Figure S6. Mass spectrum fraction 45 *Chihuahuanus coahuilae*. Figure S7. Mass spectrum fraction 50 *Chihuahuanus coahuilae*. Figure S8. Mass spectrum fraction 51 *Chihuahuanus coahuilae*. Figure S9. Mass spectrum fraction 54 *Chihuahuanus coahuilae*. Figure S10. Mass spectrum fraction 59 *Chihuahuanus coahuilae*. Figure S11. Mass spectrum fraction 29 *Chihuahuanus crassimanus*. Figure S12. Mass spectrum fraction 30 *Chihuahuanus crassimanus*. Figure S13. Mass spectrum fraction 36 *Chihuahuanus crassimanus*. Figure S14. KTxs. Sequences were obtained from the Kalium database (Kalium 2.0, Russian Science Foundation, Russia, 2019) https://kaliumdb.org/, accessed on 10 May 2023 which is a collection of natural peptides acting on potassium channels mostly originating from the venoms of animals like scorpions, snakes, spiders, and sea anemones. The alignment was made with the program Clustal Omega (EMBL’s European Bioinformatics Institute (EMBL-EBI) version 2.0.12, Cambridge, UK, 2011) https://www.ebi.ac.uk/Tools/msa/clustalo/, accessed on 10 May 2023. Figure S15. AMPs Sequences found on CAMP R3 database (Collection of Anti-Microbial Peptides, Indian Council of Medical Research (ICMR), Mumbai India, 2006). No molecular weight is reported. Table S1. Comparative view of HPLC obtained fraction, molecular weight and effects of two different scorpion venoms. Table S2. KTxs Secuence data were obtained from Kalium database (http://kaliumdb.org, accessed on 10 May 2023), which is a collection of natural peptides acting on potassium channels mostly originating from the venoms of animals like scorpions, snakes, spiders, and sea anemones. Table S3. AMPs. Scorpion-derived compounds with antibacterial activities.
