# Supplementary material for: Identification and Venom Characterization of Two Scorpions from the State of Chihuahua Mexico: Chihuahuanus coahuliae and Chihuahuanus crassimannus
Source: Toxins (Basel). 2023 Jun 27;15(7):416. doi: 10.3390/toxins15070416 (PMC10467103; doi:10.3390/toxins15070416)
Supplement: Supplementary file 1 [file toxins-15-00416-s001.zip › toxins-2414631-supplementary.pdf]

Supplementary material

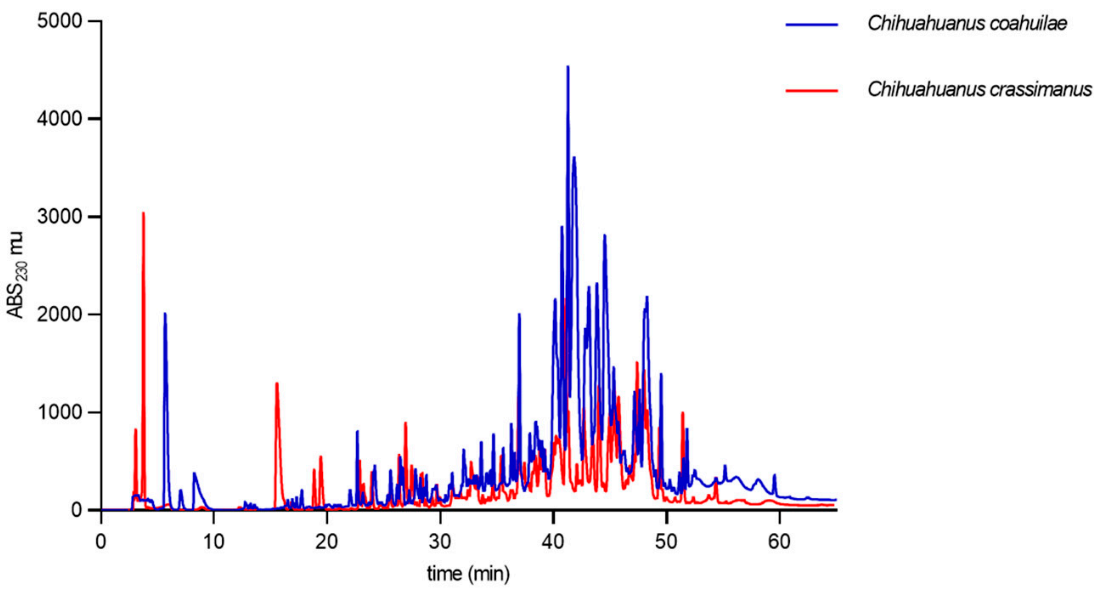

Figure S1. Overlapping HPLC profiles of two scorpion venoms. The venom of *Chihuahuanus coahuilae and Chihuahuanus crassimanus* scorpions were separated by RP-HPLC. The HPLC separation was performed using a C18 column in a gradient of 0 to 60%

Table S1. Comparative view of HPLC obtained fraction, moleculra weight and effects of two different scorpion venoms

Ch. cohahuliae venom					*Ch. crassimanus venom*					
**Fraction**	**Retention time (min)**	**Molecular mass (Da)**	**Mouse (2 μg)**	**Cricket (10 μg)**	**Fraction**	**Retention time (min)**	**Molecular mass (Da)**	**Mouse (2 μg)**	**Cricket (10 μg)**	**Comments**
**35**	36	2,977.0<br>3,066.1	-	-	**22**	37.5	3,066.7	-	-	KTx
**39**	40.7	8,302.3	Dead	-	**26**	41.3	8,302.6	-	-	NaTx
**43**	43.1	6,559.1<br>6,736.1	Dead	Dead	**29**	43.7	6,709.4<br>6,747.2	Dead	Paralyzed	Insect-Toxin / Contaminated fraction
**44**	43.6	6,643.7<br>6,616.4	-	Paralyzed	**30**	44.3	6,643	-	Paralyzed	Insect-Toxin / Contaminated fraction

Chihuahuanus coahuilae

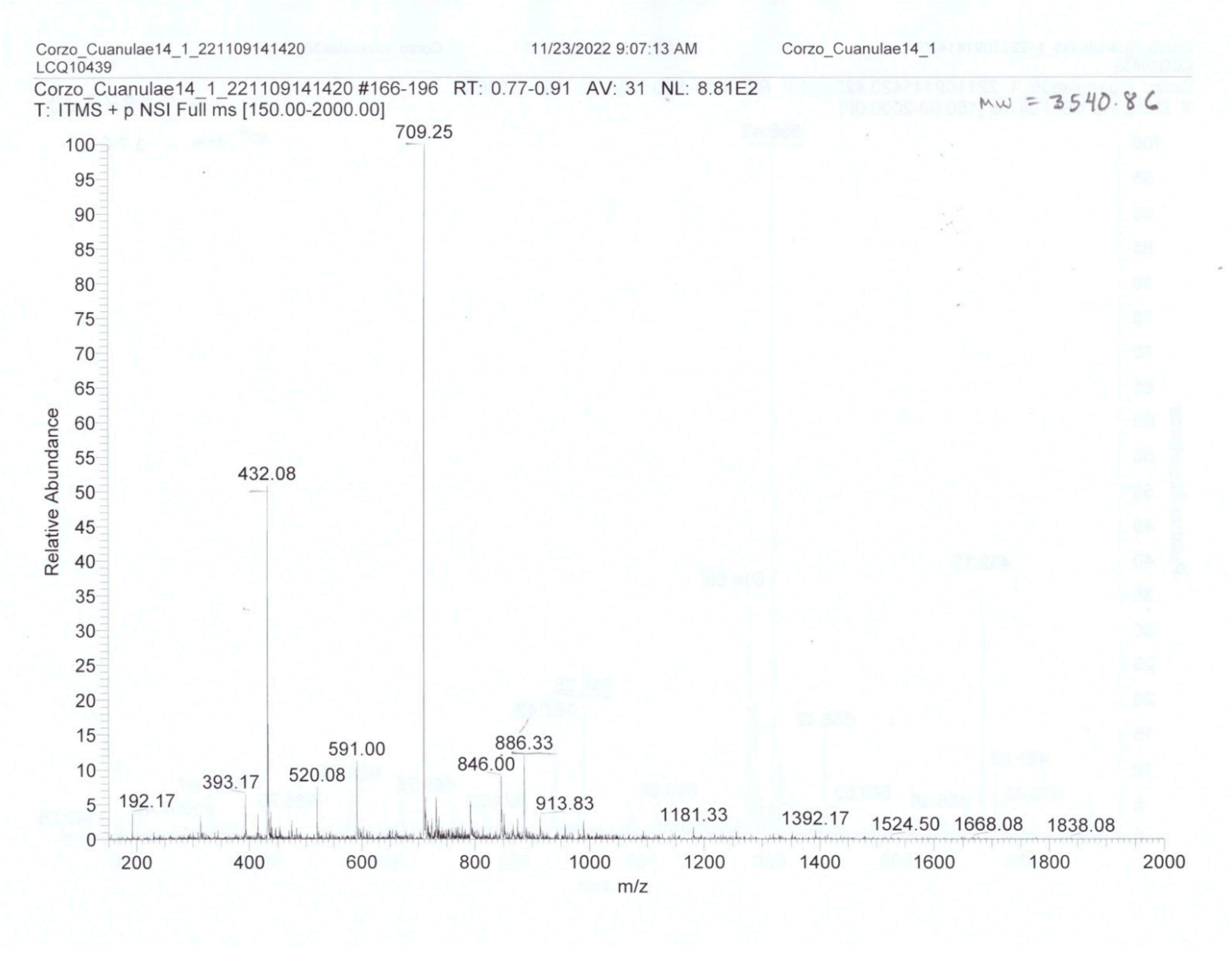

Figure S2. Mass spectrum fraction 14 *Chihuahuanus coahuilae*

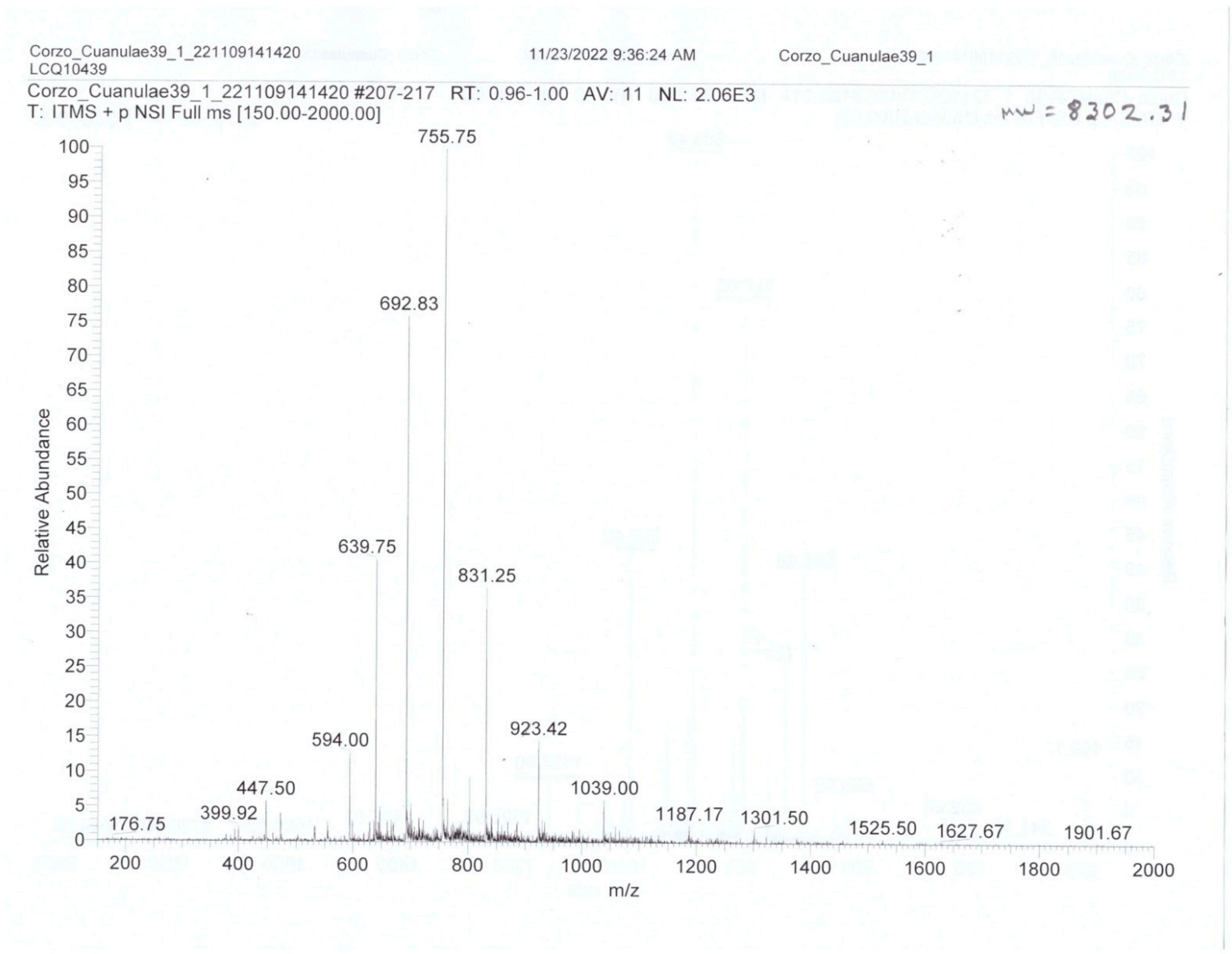

Figure S3. Mass spectrum fraction 39 *Chihuahuanus coahuilae*

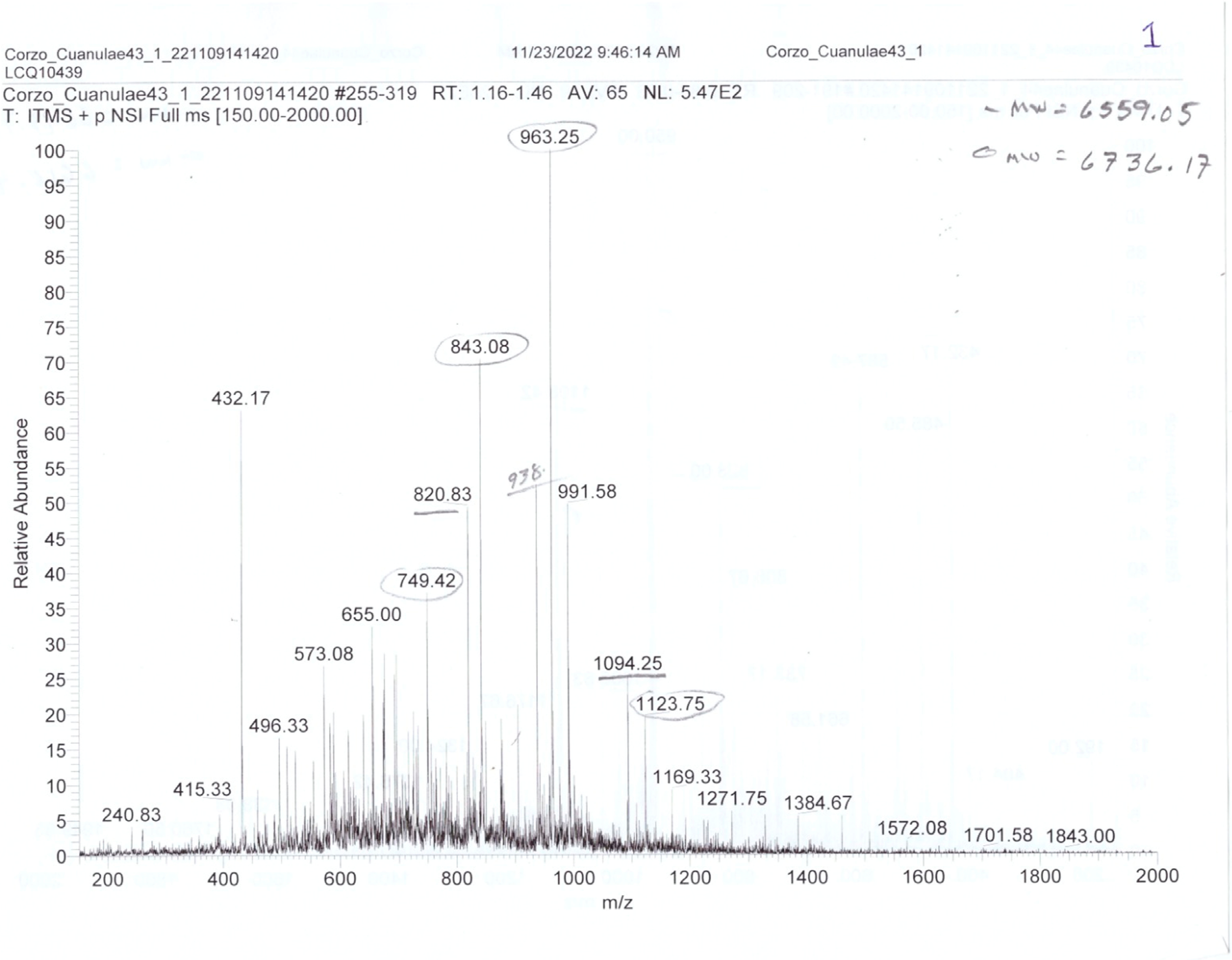

Figure S4. Mass spectrum fraction 43 *Chihuahuanus coahuilae*

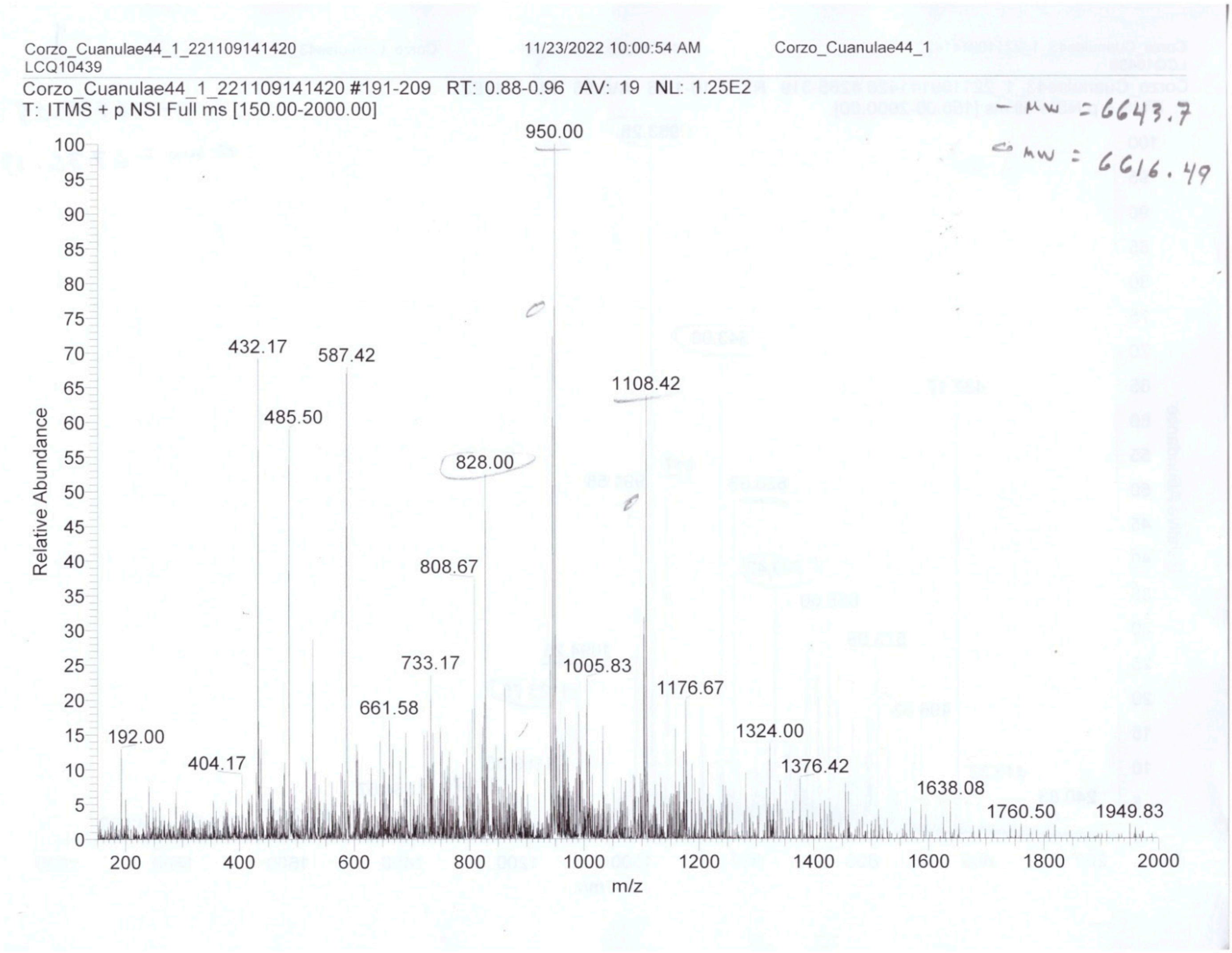

Figure S5. Mass spectrum fraction 44 *Chihuahuanus coahuilae*

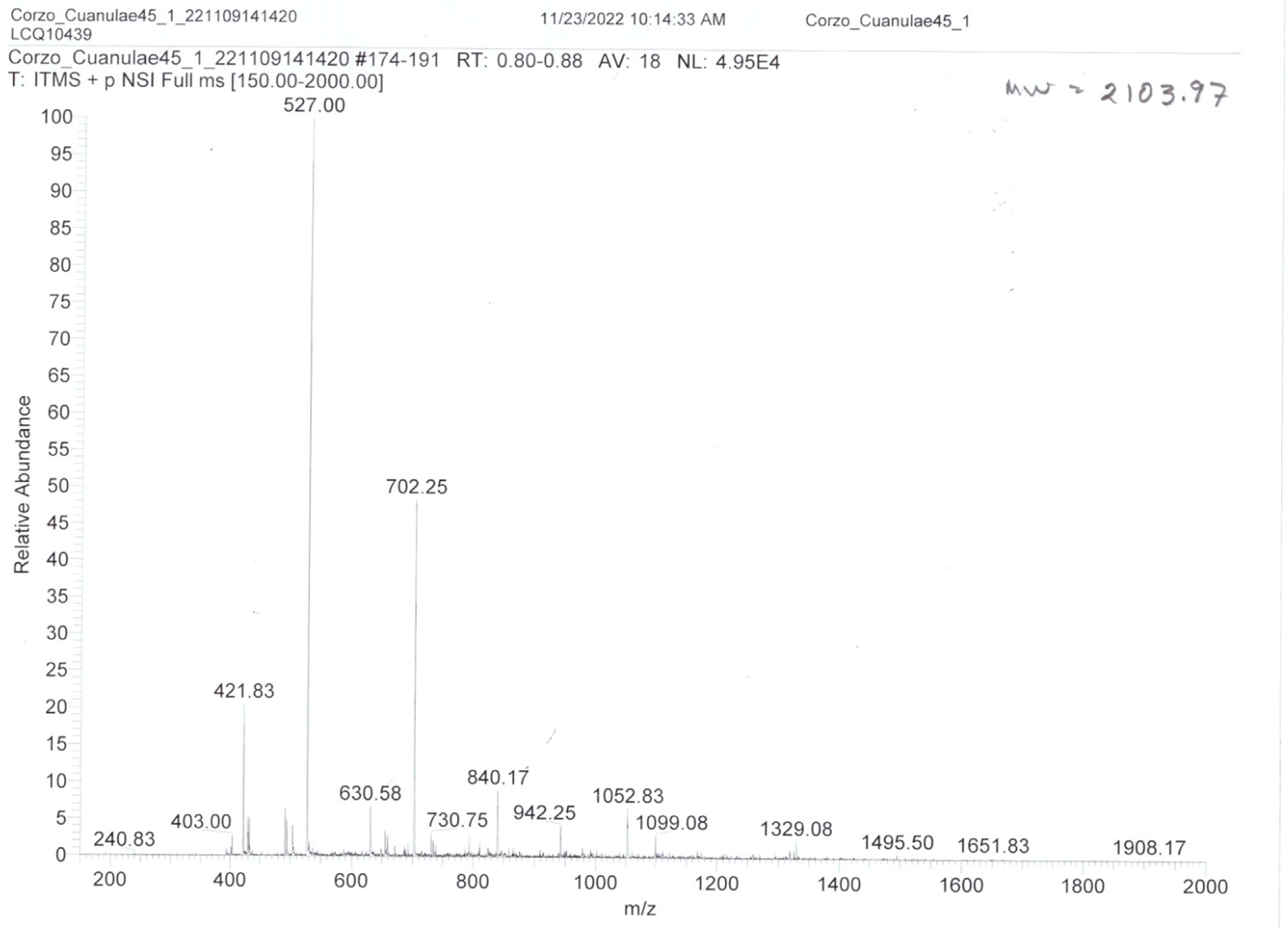

Figure S6. Mass spectrum fraction 45 *Chihuahuanus coahuilae*

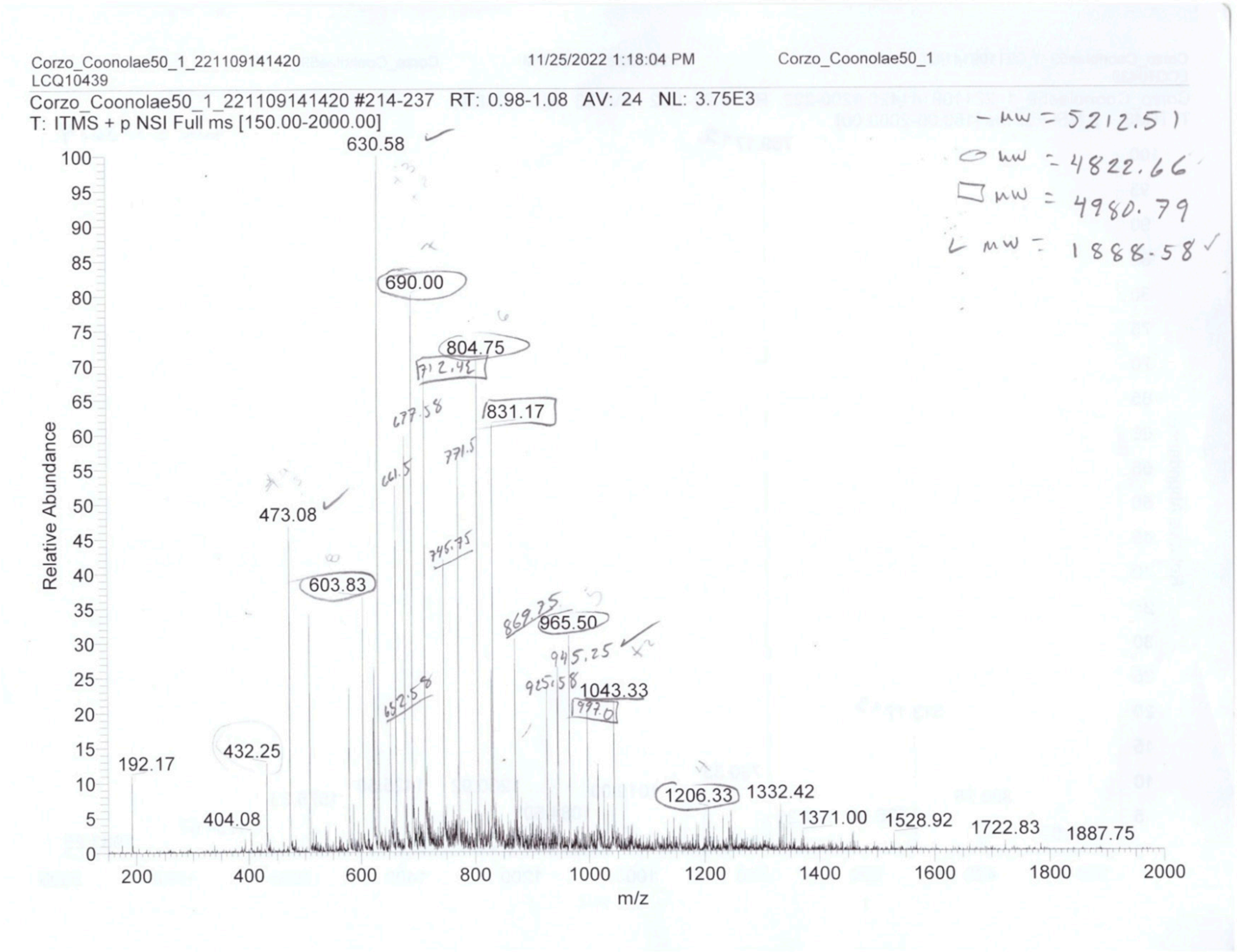

Figure S7. Mass spectrum fraction 50 *Chihuahuanus coahuilae*

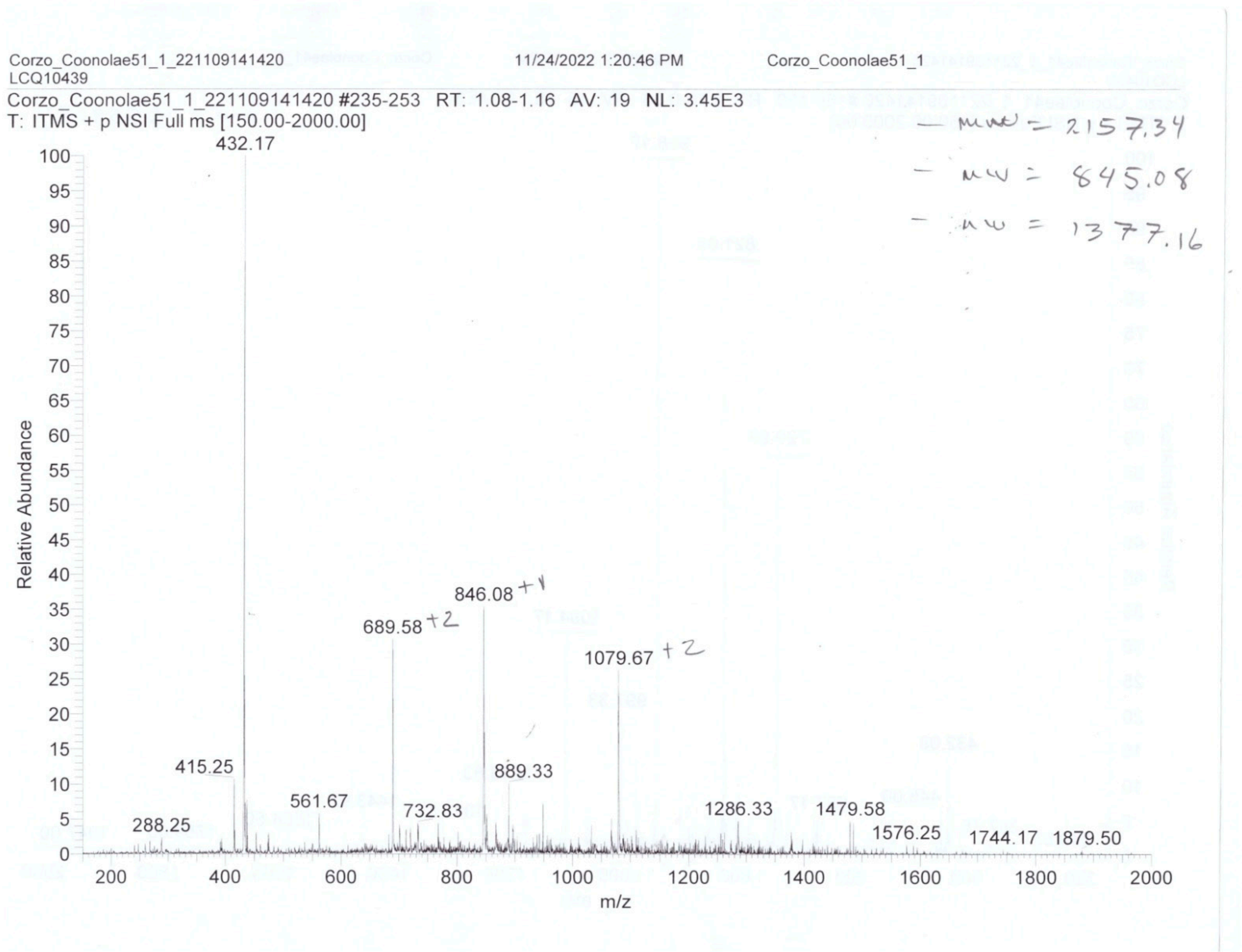

Figure S8. Mass spectrum fraction 51 *Chihuahuanus coahuilae*

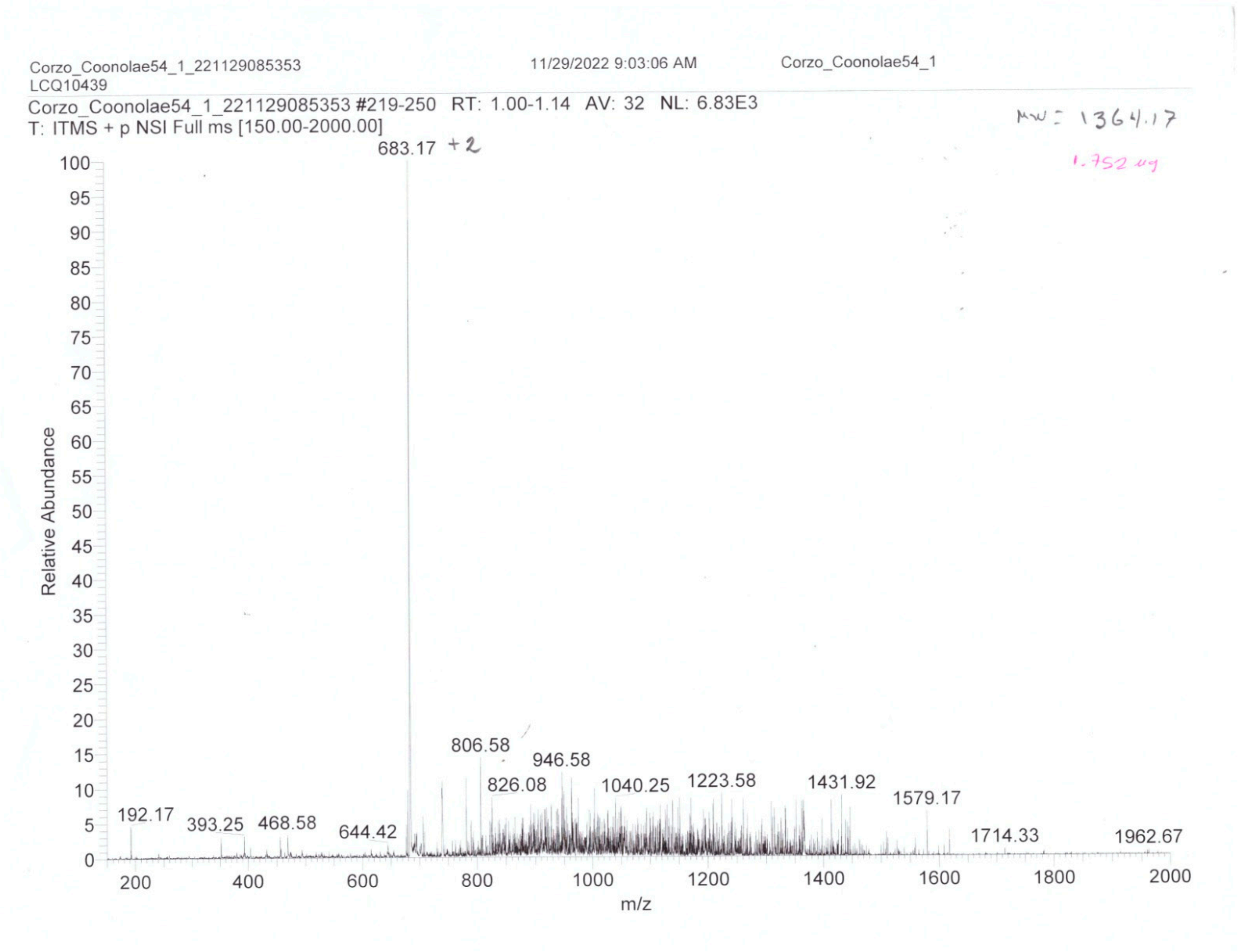

Figure S9. Mass spectrum fraction 54 *Chihuahuanus coahuilae*

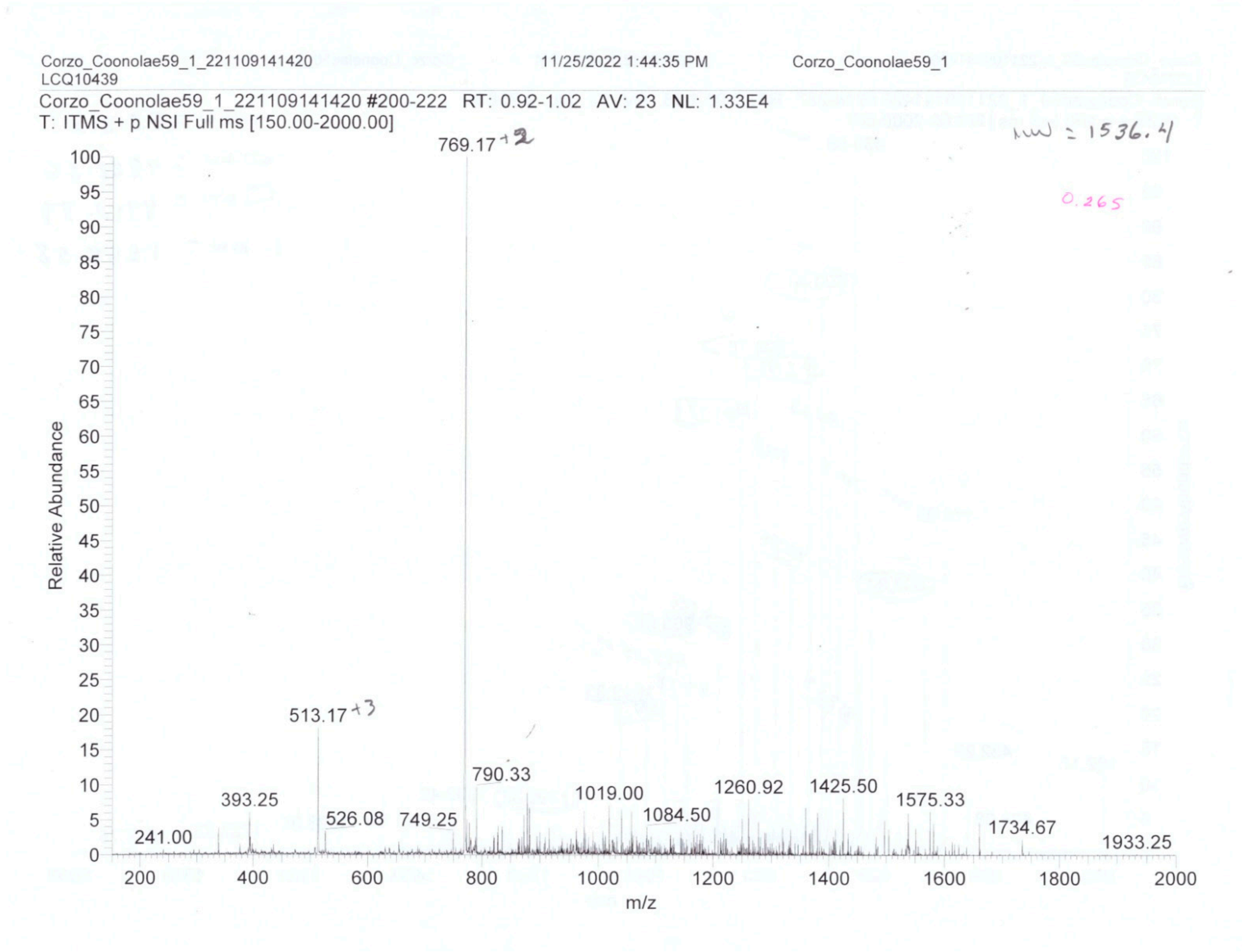

Figure S10. Mass spectrum fraction 59 *Chihuahuanus coahuilae*

Chihuahuanus crassimanus

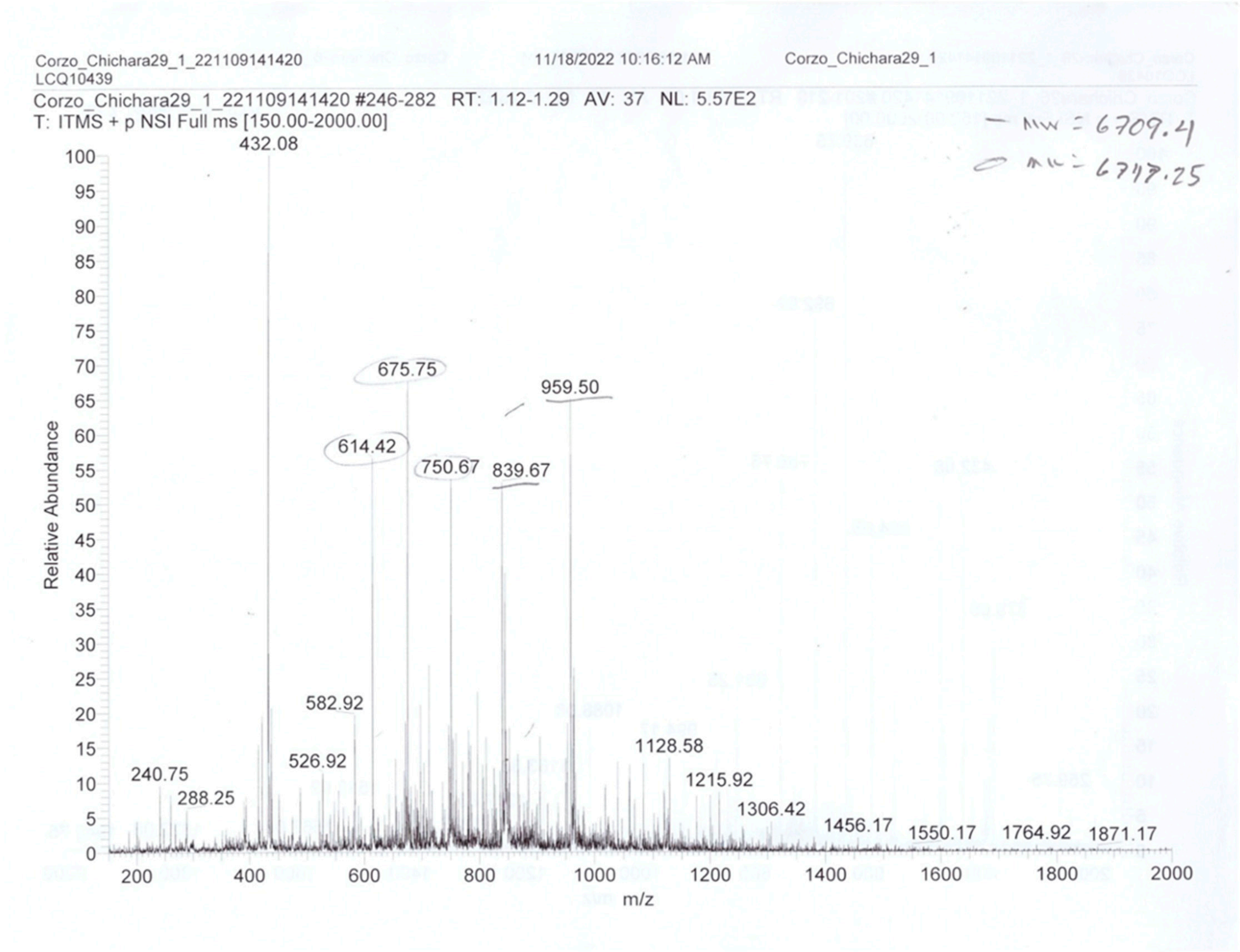

Figure S11. Mass spectrum fraction 29 *Chihuahuanus crassimanus*

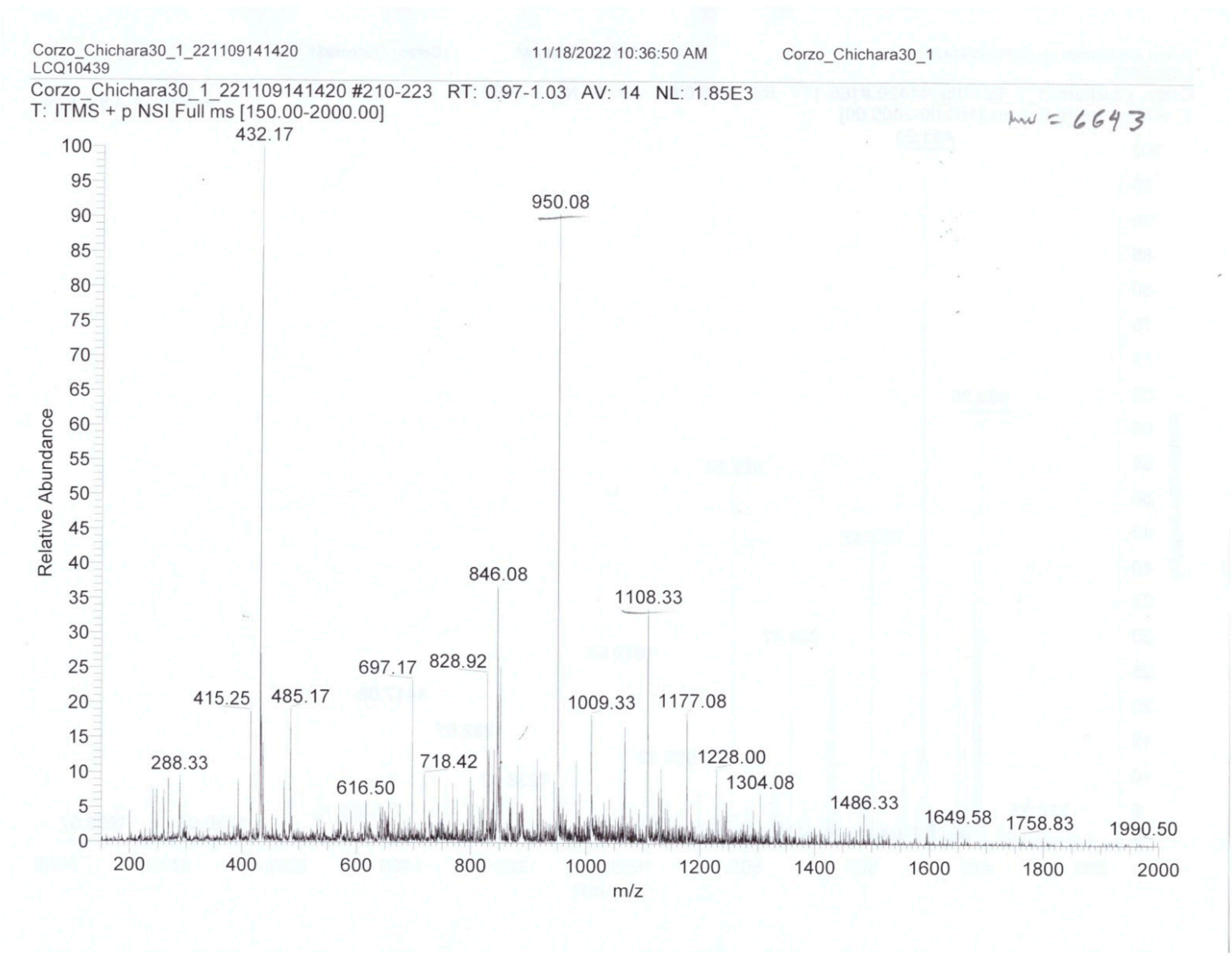

Figure S12. Mass spectrum fraction 30 *Chihuahuanus crassimanus*

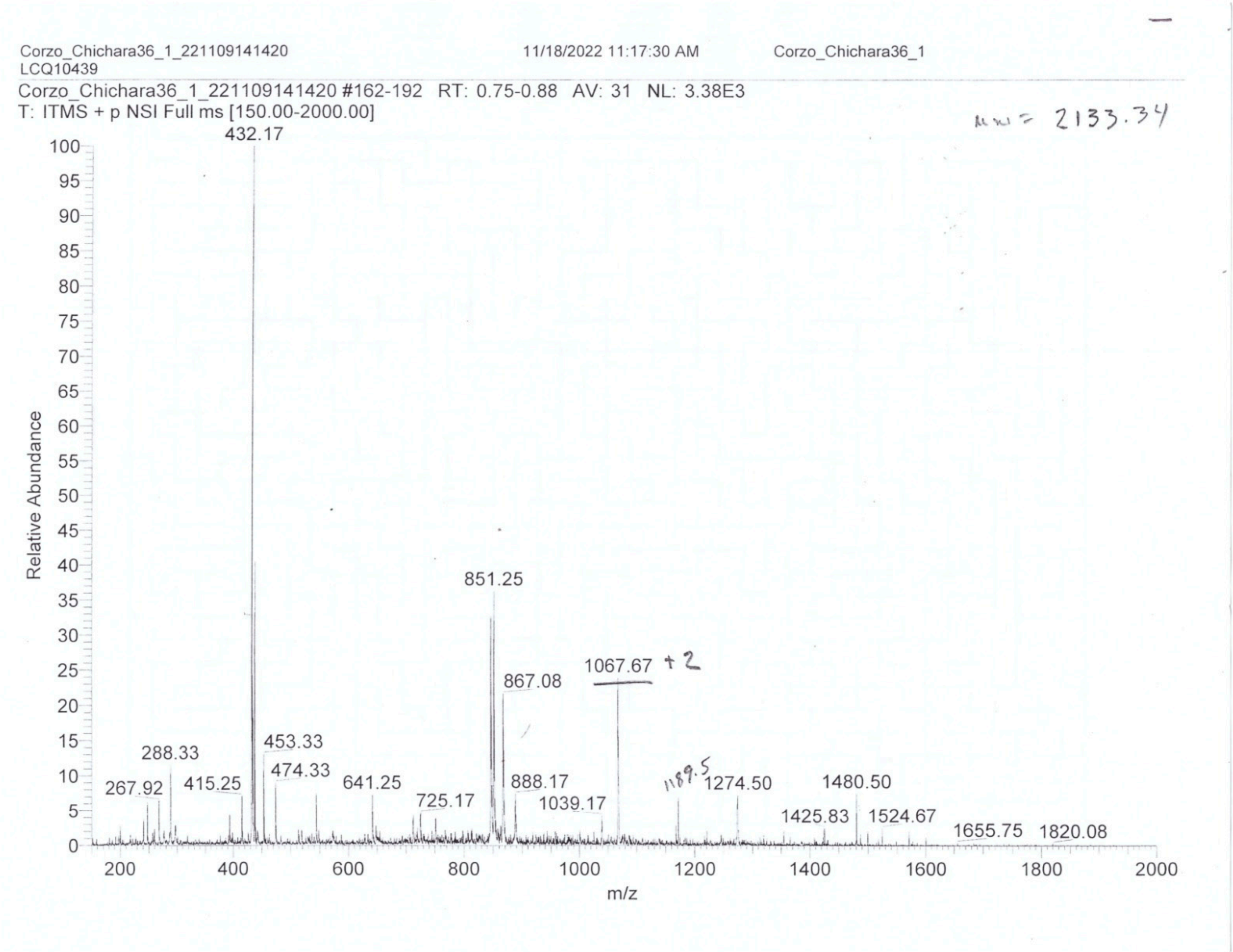

Figure S13. Mass spectrum fraction 36 *Chihuahuanus crassimanus*

```
C0HKB3_Pandinus_imperator_KTx_2.9             VDACYEACMHHHMNSDDCIEACKNPVPP-----------------    28
P0C182_C_noxius_KTx_1.11                      -----TFIDVDCTVSKECWAPCKAAFGVD-RGKCMGKKCKCYV--    37
Q9TXD1_C_noxius_KTx_2.4                       -----TIINEKCFATSQCWTPCKKAIGSL-QSKCMNGKCKCYNG-    38
P08815_C_noxius_KTx_2.1                       -----TIINVKCTSPKQCSKPCKELYGSSAGAKCMNGKCKCYNN-    39
P85529_Centruroides_suffusus_KTx_2.13         -----IFINVKCSSPQQCLKPCKAAFGISAGGKCINGKCKCYP--    38
O46028_C_noxius_KTx_10.1                      ----------AVCVYRTCDKDCK-RRGYR-SGKCINNACKCYPY-    32
P0DJ31_Vaejovis_mexicanus_smithi_KTx_23.1     ------AAAISCVGSPECPPKCR-AQGCK-NGKCMNRKCKCYYC-    36
P0DJ32_Vaejovis_mexicanus_smithi_KTx_23.2     ------AAAISCVGSKECLPKCK-AQGCK-SGKCMNKKCKCYC--    35
C0HKB2_Pandinus_imperator_KTx_24.1            ---------VAKCSTSECGHACQ-QAGCR-NSGCRYGSCICVGC-    33
P55928_Pandinus_imperator_KTx_7.2             --------TISCTNEKQCYPHCKKETGYP-NAKCMNRKCKCFGR-    35
Q10726_Pandinus_imperator_KTx_6.1             --------LVKCRGTSDCGRPCQQQTGCP-NSKCINRMCKCYGC-    35
P58498_Pandinus_imperator_KTx_6.4             ------IEAIRCGGSRDCYRPCQKRTGCP-NAKCINKTCKCYGCS    38
                                                               *    *:
```

Figure S14. KTxs. Sequences were obtained from Kalium database https://kaliumdb.org/, which is a collection of natural peptides acting on potassium channels mostly originating from the venoms of animals like scorpions, snakes, spiders and sea anemones The alignment was made with the program Clustal Omega https://www.ebi.ac.uk/Tools/msa/clustalo/.

Table S2. KTxs Secuence data was obtained from Kalium database (http://kaliu mdb.org), which is a collection of natural peptides acting on potassium channels mostly originating from the venoms of animals like scorpions, snakes, spiders, and sea anemones.

Species	Toxin	Molecular weight (Da)
Centruroides noxius	KTx 1.11	4085.97
	KTx 2.1	4195.06
	KTx 2.4	4183.048
	KTx 10.1	3731.476
Vaejovis mexicanus smithi	KTx 23.1	3863.776
	KTx 23.2	3667.602
Pandinus imperator	KTx 6.1	3834.648
	KTx 6.4	4180.032
	KTx 7.2	4065.8637
	KTx 24.1	3334.7009
	KTx 2.9	3126.4445
Centruroides suffusus	KTx 2.13	4000.94

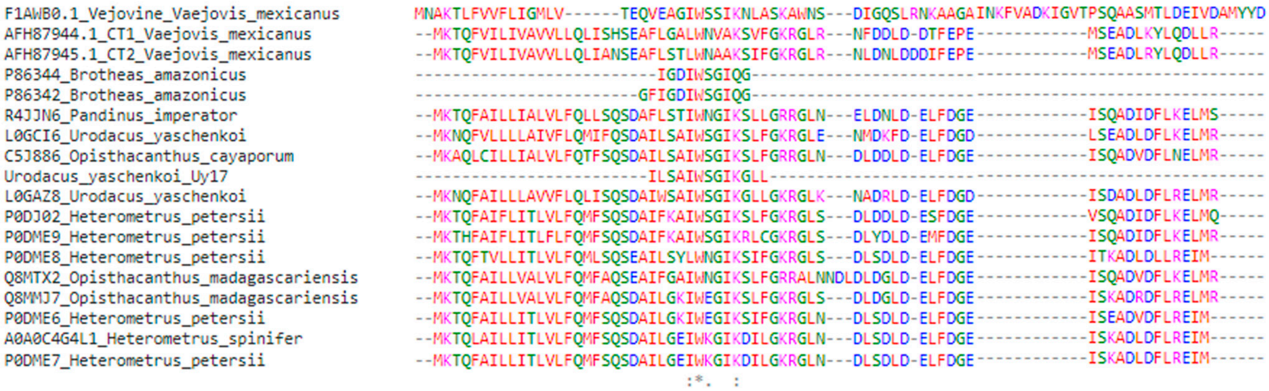

Figure S15. AMPs Sequences found on CAMP R3 database- No molecular weight its reported.

Due to their wide range of functions and targets, rapid efficacy, and low potential for inducing resistance, antimicrobial peptides (AMPs) have emerged as promising candidates for new antibiotics, as highlighted by Huang et al. (2010) (Huang et al., 2010). AMPs primarily eliminate microorganisms by employing mechanisms that target their membranes and form pores, which inherently pose greater challenges for microbes to develop resistance against, as discussed by Hancock and Sahl (2006) (Hancock & Sahl, 2006). A considerable portion of the characterized scorpion venom Non-Disulfide-Bridged-Peptides (NDBPs) exhibit antimicrobial properties, thus classifying them as AMPs, as noted by Harrison et al. (2014) (Harrison et al., 2014). The precise reason for their presence in scorpion venom remains unclear. They may potentially contribute synergistically to venom activity or serve as a component of the venom gland's antimicrobial response, as suggested by Kuhn-Nentwig (2003) (Kuhn-Nentwig, 2003).

Table S3. AMPs. **Scorpion-derived compounds with antibacterial activities**

Specie	Antibacterial agent	Molecular weight	Reference
U. yaschenkoi	Uy234	1986.19	(Cesa-Luna et al., 2019)
	Uy17	1369.43	
	Uy192	1459.98	
D. melici	Red 1,4-benzoquinone: 3,5-dimethoxy-2-(methylthio) cyclohexa-2,5-diene-1,4-dione<br>Blue 1,4-benzoquinone: 5-methoxy-2,3-bis(methylthio)cyclohexa-2,5-diene-1,4-dione	168.15	(Carcamo-Noriega et al., 2019)
Hadrurus gertschi)	Hadrurin	4435.3	(Torres-Larios et al., 2000)
Opisthacanthus madagascariensis	IsCTs	1501.9	(Dai et al., 2001)
Vaejovis mexicanus	vejovine	4873	(Hernández-Aponte et al., 2011)
Pandinus imperator	Scorpine	8350	(Conde et al., 2000)
	Pandinins 2	2612.11	(Corzo et al., 2001)
